# Targeted mutagenesis in the medicinal plant *Salvia miltiorrhiza*

**DOI:** 10.1038/srep43320

**Published:** 2017-03-03

**Authors:** Bin Li, Guanghong Cui, Guoan Shen, Zhilai Zhan, Luqi Huang, Jiachun Chen, Xiaoquan Qi

**Affiliations:** 1 Hubei Key Laboratory of Natural Medicinal Chemistry and Resource Evaluation, School of Pharmacy, Tongji Medical College, Huazhong University of Science and Technology, Wuhan 430030, China; 2 Key Laboratory of Plant Molecular Physiology, Institute of Botany, Chinese Academy of Sciences, Beijing 100093, China; 3 National Resource Center for Chinese Materia Medica, China Academy of Chinese Medical Sciences, Beijing, 100700, China

## Abstract

CRISPR/Cas9 is a powerful genome editing tool that has been extensively used in model plants and crops, such as *Arabidopsis thaliana*, rice, wheat, and soybean. Here, we report the use of CRISPR/Cas9 to precisely knock out the committed diterpene synthase gene (*SmCPS1*) involved in tanshinone biosynthesis in *Salvia miltiorrhiza*, a traditional Chinese medicinal herb with significant pharmacological activities, such as vasorelaxation, protection against ischemia-reperfusion injury, and antiarrhythmic effects. Three homozygous and eight chimeric mutants were obtained from 26 independent transgenic hairy root lines by *Agrobacterium rhizogenes*-mediated transformation. The metabolomic analysis based on LC-qTOF-MS and Q-TRAP-LC-MS/MS revealed that tanshinones, especially cryptotanshinone, tanshinone IIA and tanshinone I, are completely missing in homozygous mutants, without influencing other phenolic acid metabolites. By contrast, tanshinones are decreased but still detectable in chimeric mutants, which is similar to a previously-reported an RNAi study of *SmCPS1*. These results demonstrate that *Agrobacterium rhizogenes-* mediated transformation using CRISPR/Cas9 is a simple and efficient genome editing tool in *S. miltiorrhiza*, thus paving the way for large-scale genome editing in *S. miltiorrhiza*, which is important for pathway elucidation of secondary metabolites, quality improvement, and yield increases for this valuable traditional Chinese medicinal herb.

CRISPR (clustered regularly interspaced short palindromic repeats) associated proteins act as a part of the pathogen defense system employed by certain bacteria and archaea. The host cell acquires its resistance to a pathogenic organism by incorporating fragments of a pathogens DNA into its CRISPR loci, and subsequently uses the transcribed CRISPR RNA (crRNA) to guide the degradation of pathogen transcripts^1^,^2^. The core of a type II CRISPR/Cas system from *Streptococcus pyogenes* is a complex between Cas9 nuclease, crRNA and trans-activating crRNA (tracrRNA), which first binds to and then cleaves homologous sequences in the pathogen. The short protospacer adjacent motif (PAM), downstream of the target sequence is needed for binding and cleavage of Cas9 nuclease. Different PAMs can be recognized by different kinds of Cas9 nuclease. For *Streptomyces pyogenes* Cas9 (spCas9), a ‘NGG’ sequence is recognized specifically as a PAM. The function of Cas9 nuclease is to generate double-stranded breaks, which potentially disrupt pathogen gene function either by leading to indel formation after non-homologous end joining (NHEJ) that might result in a premature stop or by inducing homologous recombination with exogenous DNA repair templates supplied^3^.

The plant genome editing technology based on the type II CRISPR/Cas system relies on *Agrobacterium tumefaciens* transformation technology to establish mutants for gene function analysis in many plants species, such as *Arabidopsis thaliana*, rice, and wheat^3^,^4^,^5^,^6^, and has also recently been successfully combined with *A. rhizogenes*-mediated transformation in tomato and soybean^7^,^8^. *A. rhizogenes*-based Ri transformed hairy roots show the characteristics of rapid growth, reduced apical dominance, high branching, and enhanced stable production of secondary metabolites, making them potentially attractive as a promising system for investigating biosynthesis of various secondary metabolites, especially in medicinal plants^9^,^10^,^11^,^12^,^13^. For example, the hairy root culture system induced by CRISPR/Cas9 could be utilized in synthetic biology as a bioreactor to produce certain bioactive medicinal compounds, such as taxol, a diterpene isolated from the *Taxus brevifolia* Pacific yew tree with the activity of anticancer^14^. Unfortunately, it is relatively difficult to generate and culture hairy roots of *Taxus brevifolia*.

*Salvia miltiorrhiza*, a diploid species belonging to the family *Lamiaceae* that is native to some regions of China and is highly prized in Chinese herbal medicine, is enriched in diterpene compounds. Its pharmacological activity is largely due to the presence of the lipid-soluble compounds known as tanshinones, along with the water-soluble phenolic acids, including rosmarinic acid, salvianolic acid, and lithospermic acid^15^. The tanshinones have been shown to promote blood circulation and antiphlogosis, while salvianolic acid has demonstrated efficacy as a treatment for some cardiovascular ailments and acts as a protectant against ischemia-reperfusion injury to the brain^16^. Tanshinones are specifically accumulated in the periderm of the reddish roots of *S. miltiorrhiza*^17^,^18^. The predominant tanshinones in this species include: cryptotanshinone, tanshinone IIA, and tanshinone I (Fig. 1). As tanshinones have the same precursor (GGPP) that is required for taxol biosynthesis, a knock-out of the post-GGPP synthesis step in the tanshinones biosynthesis pathway might promote the accumulation of the substrate for taxol synthesis, which could be exploited by, for example, adding a cluster of genes involved in the taxol pathway to roots.

Two diterpene synthases, SmCPS1 and SmKSL1, have been shown to cyclize the diterpenoid precursor (*E, E, E*)-geranylgeranyl diphosphate into the tricyclic olefin miltiradiene, the reaction that establishes the abietane-type stereochemistry of tanshinones^19^. Subsequently, three *CYP76* subfamily genes, *CYP76AH1*^13^, *CYP76AH3*^20^, and *CYP76AK1*^20^, transform miltiradiene to seven oxygenated diterpenoids (Fig. 1). Another study used RNAi of *SmCPS1* to provide molecular genetic evidence that this gene is involved in the tanshinone biosynthetic pathway^17^,^21^. The present report illustrates the feasibility of using CRISPR/Cas9 to generate a knock-out of *SmCPS1* in *S. miltiorrhiza*. Further, we demonstrate the success of the strategy to successfully block the metabolic flux through GGPP to tanshinone, highlighting the potential for switching GGPP to valuable diterpenes like taxol.

Our present study also paves the way for large-scale genome editing in *S. miltiorrhiza*. The *S. miltiorrhiza* genome harbors a large number of genes encoding terpene synthases and P450 proteins of unknown function^13^,^15^,^18^,^22^,^23^,^24^. Although RNAi has been proven as a highly-effective approach for identifying the enzymes responsible for particular steps of various secondary metabolite synthesis pathways^17^, a high frequency of non-specific gene silencing can occur wherein sequence homology promotes the degradation of non-target transcripts^25^. Using the CRISPR/Cas9 system as a gene knock-out technology is thought to be less likely to suffer from this problem, because the target sequence is only 20 bp and is designed uniquely^6^. It is also noteworthy that we used plants of a fifth generation *S. miltiorrhiza* inbred line as explants; this ensured that only one allele of a given target locus exists in the wild type background.

## Results

### CRISPR/Cas9 vector construction

**Figure 2:**
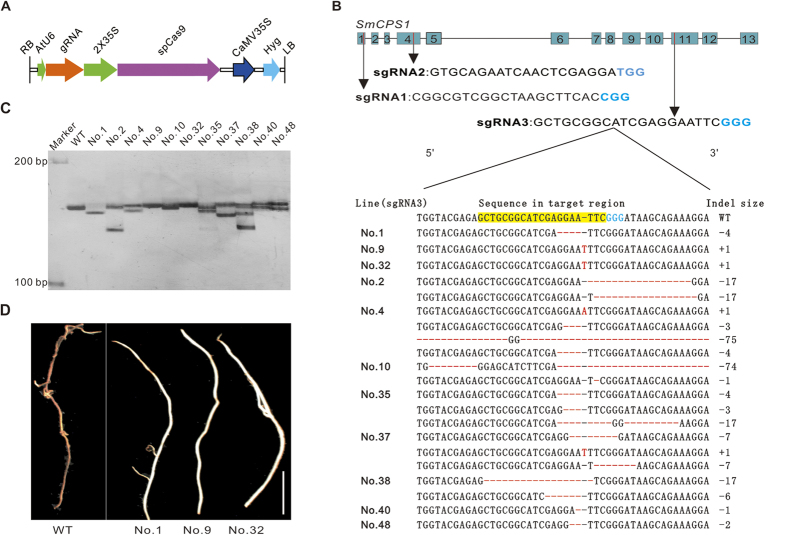
Targeted genome modification using the CRISPR/Cas9 system. (**A**) The Cas9 expression cassette is driven by the 2X35S promoter. The gRNA is driven by the *A. thaliana* U6-26 promoter. The hygromycin resistance gene is driven by CaMV 35S. (**B**) Upper: Gene structure of *SmCPS1* and the location of three gRNAs. Numbers 1–13 indicate exons. The location of sgRNA1, sgRNA2, and sgRNA3 are indicated by red lines and by black arrows in exons 1, 4, and 11. “CGG”, “TGG”, and “GGG” sequences are, respectively, the PAMs of sgRNA1, sgRNA2, and sgRNA3. Lower: Sequences amplified from genomic DNA isolated from primary transgenic hairy root lines of sgRNA3 derivatives. The WT sequence appears at the top, with the PAM sequence shown in blue and the target sequence highlighted in yellow. DNA insertions, point mutations are shown as red letters. Deletions are shown as dashes: + : insertion, −: deletion. The indel size shows the loss/gain in amplicon length in target loci. The WT sequence is not listed for each line. (**C**) Denaturing PAGE separation. A silver-stained denaturing PAGE separation showing the DNA fragments generated from WT (wild type) and different mutant root lines. M: 100 bp DNA marker. (**D**) The appearance of wild type and homozygous mutants (No. 1, No. 9, No. 32) roots. The WT produces a red periderm while No. 1, No. 9, and No. 32 are white. Bar = 1 cm.

Here, we tested the CRISPR/Cas9 system that has been successfully applied in *Arabidopsis thaliana*^3^ to see whether it can also effectively induce gene-specific modification in *S. miltiorrhiza*. Briefly, both a nuclear localization signal and a Flag tag were appended to the amino and carboxyl termini of spCas9 to ensure the deposition of spCas9 in the nucleus. The spCas9 expression cassette was driven by the CaMV 35S promoter. A vector harboring a single chimeric guide RNA (sgRNA), formed from a fusion between crRNA and tracrRNA, was driven by the *Arabidopsis thaliana* U6-26 promoter (Fig. 2A)^3^.

Many online tools for sgRNA design have been developed, and these can be used to score and rank uploaded draft sequences (http://crispr.mit.edu/, http://www.broadinstitute.org/mpg/crispr_design/, http://www.e-crisp.org/E-CRISP/etc.). All of those tools are based on the whole genome sequencing of a given species. However, the genome sequence data for *S. miltiorrhiza* has not yet been incorporated in such online tools, so we manually designed/selected three sgRNAs based on our raw DNA sequence data^18^. We wrote a R program to investigate off-target effects (Data S1). The sgRNA sequence containing four base mismatches was searched over the genome assembly. Thereafter, three mutation targets were chosen: one at the first, fourth, and eleventh exons of *SmCPS1*. Each of the 20 bp target sequences was designed upstream of the aforementioned ‘NGG’, the spCas9 PAM (Fig. 2B).

With a two-step assembly strategy, the three target sequences were initially inserted into an sgRNA expression cassette, and were then combined with a Cas9 expression cassette in the pCambia1300 plant binary vector (Cambia, Australia) (Figure S1). A number of independent transgenic hairy root cultures were produced for each target (20 root lines for sgRNA1, 24 lines for sgRNA2, and 26 lines for sgRNA3, see Table S1) using *A. rhizogenes* strain ACCC10060, in which a CRISPR/Cas9 plasmid was transformed.

### Silver-stained denaturing PAGE separation for detection of mutants of *SmCPS1*

Single-strand DNA fragments of various lengths are readily recognized in silver-stained denaturing PAGE separation; these single-strand DNA fragments can be thusly separated at one-base resolution. It is highly suitable for the detection of new alleles induced by CRISPR/Cas9, and does not require any intermediate restriction enzyme digestion step. Only sgRNA3 generated a variety of DNA profiles (Fig. 2C). As only one *SmCPS1* allele exists in these fifth generation *S. miltiorrhiza* inbred plants, a single DNA fragment present in WT root lines transformed with a control plasmid (CRISPR/Cas vector without spacer sequence), as was also the case for both successfully-target homozygous mutants and for off-target lines (No. 1, No. 9, and No. 32 in Fig. 2C, off-target lines are not shown in this figure). Because multiple alleles exist in chimeric mutants, their PAGE profiles showed more than one DNA fragment, indicating the heteroalleles resulting from different mutation events^26^. Although not all of the heteroalleles could be screened on gels, silver-stained denaturing PAGE separation is still an efficient method to identify mutants prior to sequencing.

### Genotyping and phenotyping of mutants

The sequencing data also showed the same result: no mutant allele could be detected in lines transformed with sgRNA1 or sgRNA2, reinforcing the desirability of selecting multiple targets^27^. Among the sgRNA3-transformed root lines, only 3 of a total of 18 lines that showed only one DNA band (line No. 1, line No. 9, and line No. 32) were found to be homozygous mutants (Fig. 2B and C). Following the cloning of PCR amplicons from independent hairy root lines showing multiple DNA bands in silver-stained denaturing PAGE separation, twenty-four independent colonies were selected and sequenced for each line. We obtained three homozygous mutants and eight chimeric mutants from 26 transgenic lines out of a total of 46 transformed lines. The proportion of mutants was estimated to be 11.5% and 30.8%, respectively, for the homozygous and chimeric mutants (Table S1). In contrast to the red appearance of wild type roots, the roots of homozygous mutants were white (Fig. 2D).

### Tanshinone biosynthesis is disrupted in *SmCPS1* homozygous mutants

**Figure 3:**
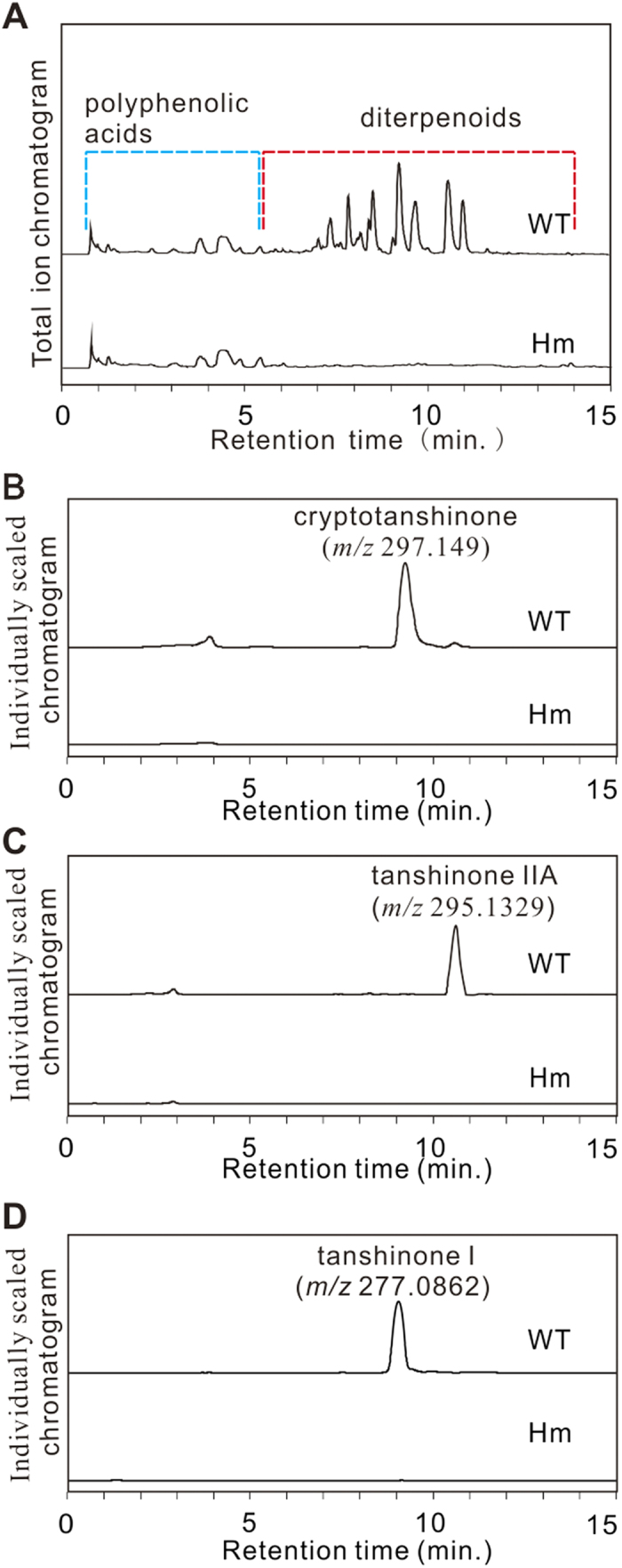
UPLC-ESI-qTOF-MS analysis. (**A**) Total ion chromatogramof homozygous mutants (Hm) and wild type (WT). (**B**–**D**) Extracted ion chromatogram of cryptotanshinone (*m/z* 297.1490), tanshinone IIA (*m/z* 295.1329), and tanshinone I (*m/z* 277.0862). The lack of the three predominant tanshinones in Hm indicates that homozygous mutants hardly generate these compounds.

A previously-established UPLC-ESI-qTOF-MS method^17^ was used to identify the alterations of metabolites in the homozygous mutants. Multiple ion signals were observed within the window from a retention time (RT) of 5.7 min to 12.0 min in wild type (WT) hairy root; these signals are known to represent diterpenoid tanshinones^16^. The mutants had nearly no signal representative of these compounds (Fig. 3A). In contrast, the polyphenolic acid profiles of the wild type and the mutant were very similar to each other. The three predominant tanshinones, including cryptotanshinone (RT 9.29 min, *m/z* 297.1490), tanshinone IIA (RT 10.62 min, *m/z* 295.1329), and tanshinone I (RT 9.13 min, *m/z* 277.0862) were all absent from the mutants (Fig. 3B–D).

**Figure 4:**
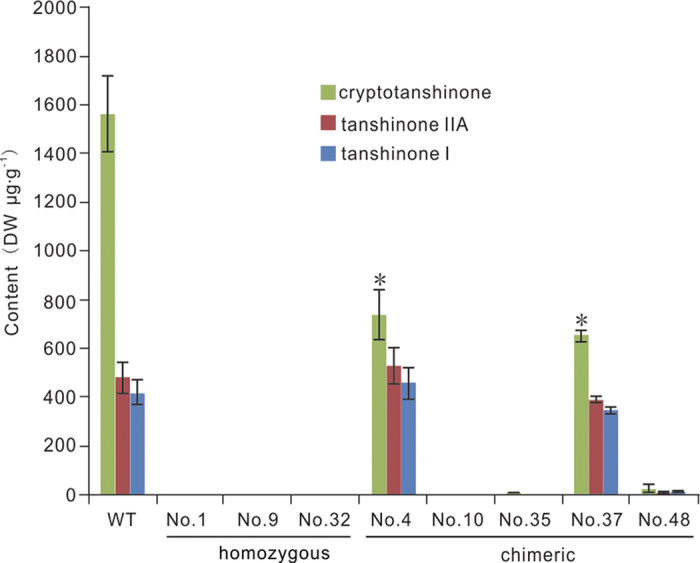
Quantitative Q-TRAP-LC-MS/MS analysis of the three predominant tanshinones in the mutants. The content of the three predominant tanshinones (cryptotanshinone, tanshinone IIA, and tanshinone I) in wild type (WT) roots, the roots of homozygous mutant lines (No. 1, No. 9, and No. 32), and chimeric lines (No. 4, No. 10, No. 35, No. 37, and No. 48). Values represent the average of three trials, with error bars representing the standard deviation.

A quantitative Q-TRAP-LC-MS/MS analysis was further used to confirm the disruption of tanshinone biosynthesis in the mutants. None of the mutants accumulated any of the three predominant tanshinones (Fig. 4), suggesting that knocking out of *SmCPS1* had specifically disrupted the tanshinone biosynthesis pathway, a finding in agreement with the results of a previous study that used RNAi to silence the expression of *SmCPS1*^17^.

### Chimeric mutants also had reduced tanshinone content

Five chimeric mutants, all of which had similar growth rates compared with wild type cultures, were analyzed by quantitative Q-TRAP-LC-MS/MS to explore the influence of the chimeric mutations of *SmCPS1* on the three predominant tanshinones. As with homozygous mutants, the chimeric mutant line No. 10 lacked all three tanshinones; it was the only chimeric mutant line with this exact phenotype (Fig. 4). All 24 sequenced clones from line No. 10 harbored no WT sequence; rather, it had two distinct mutated alleles, a 1 bp deletion or a 74 bp deletion (Fig. 2B and Table S2). Other chimeric mutants also exhibited reduced tanshinone content (Fig. 4). In lines No. 35 and No. 48, the content of the three predominant tanshinones was greatly reduced. In lines No. 4 and No. 37, the content of both tanshinone I and tanshinone IIA was comparable to that in the wild type, but of the content of cryptotanshinone was reduced by about one half. These results revealed that the expression of the *SmCPS1* gene was knocked-down, not knocked-out, in chimeric mutants, unlike the complete knock-out of the homozygous mutants.

## Discussion

A previous study showed that *SmCPS1* knock-down could generate mutants with obvious tanshinone-related phenotypes (white root and tanshinones reduction)^17^. With this knowledge, we selected the *SmCPS1* gene as the target for an implementation of a CRISPR/Cas9-based gene modification. Both the previous and the present study showed that *SmCPS1* mutants provide strong evidence for the association between *SmCPS1* function and tanshinone accumulation^24^. This effective approach for generating designed modifications of the *S. miltiorrhiza* genome paves the way for the rapid characterization of the as many as tens of thousands of the genes of this species, including for example genes associated with the biosynthetic pathways of tanshinone or other water-soluble caffeic acid-derived phenolic acids. One can imagine that CRISPR/Cas9-based gene modification will be especially useful in studies of multi-gene families such as the *P450s*. This promises to aim efforts to improve both quality and yield in this valuable medicinal herb.

In this study, we obtained 26 independent transgenic lines. The percentage of the independent T_0_ transgenic *S. miltiorrhiza* with successful mutations in *SmCPS1* was about 42.3%. This included three homozygous mutant lines and eight chimeric mutant lines (Table S1). A possible explanation for the relatively high number of chimeric hairy root lines is that spCas9 may have introduced different mutations in diverse types of cells. Despite the presence of the chimeric root lines from our initial mutagenesis, the three homozygous mutants have, after more than one year of subculturing, exhibited no change in their tanshinone production phenotype. These stable tanshinone-free mutants are of interest in themselves, as their lack of tanshinones simplifies the isolation of valuable compounds like salvianolic acid B for further large scale production efforts. Moreover, the *SmCPS1* mutant in which the GGPP is accumulated^17^ could in theory serve a source of other valuable diterpenes such as taxol, if synthetic biology approach were to be applied.

**Figure 5:**
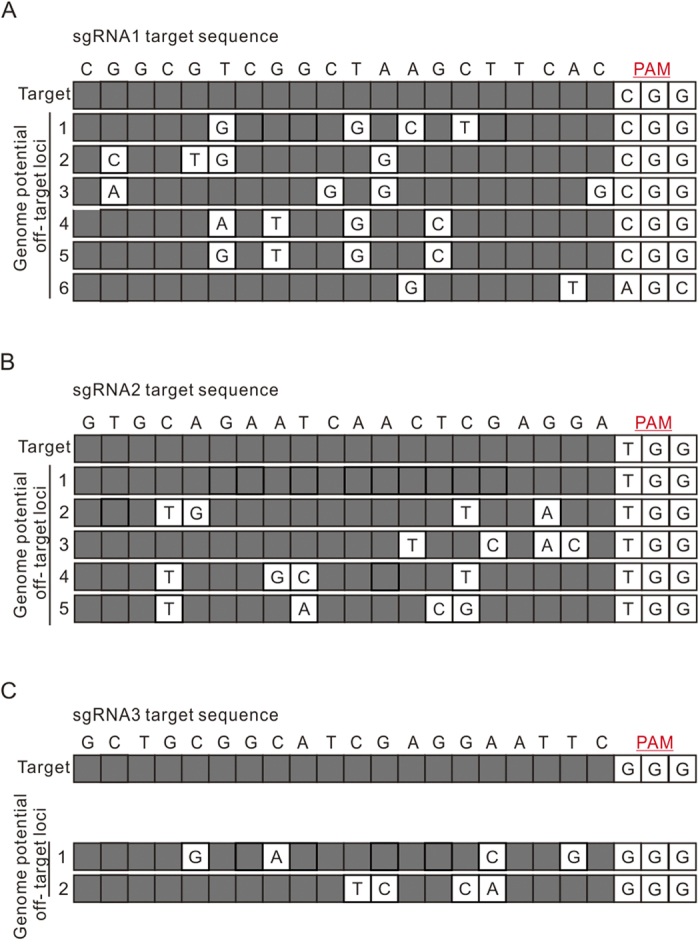
Lists of potential off-target loci for gRNAs used in this study. (**A**–**C**) Sequence alignment for sgRNA1, sgRNA2, and sgRNA3. Rows represent potential off-target loci in the *S. miltiorrhiza* genome; mismatches are shown in white cells. Grey cells denote same nucleotides compared with the target.

*Arabidopsis thaliana* is a model dicot from which many promoters are known to also function in other dicots species. In this study, we tested the use of *Arabidopsis thaliana* optimized spCas9 and its AtU6 promoter to selectively modify the *S. miltiorrhiza* genome. However, only one out of the three designed sgRNAs induced modifications; it induced a modification rate of 42.3%. Our off-target analysis results showed that six similar sequences with four-base mismatches were found compared with sgRNA1 (Fig. 5A). It is known that the off-target efficiency of a given sgRNA is largely reduced when there are four-base differences in similar sequences. Given this, the off-target activity of sgRNA1 transformation events may result from the un-optimized CRISPR vector for *S. miltiorrhiza* that we used here^28^. For sgRNA2, four similar sequences with four-base mismatches were found including a same sequence in other loci of the genome (Fig. 5B). If the same sequence was found at other loci, they could lead to off-target modifications, and this may be exacerbated by the un-optimized CRISPR vector. For sgRNA3, only two similar sequences, with four-base differences were found. Regardless of the fact that sgRNA3 induced modifications, its mutation rate was only 42.3% (Fig. 5C). Considering this, perhaps *S. miltiorrhiza* optimized CRISPR/Cas9 vectors should be constructed in further work, including a codon optimized Cas9 as well as use of a native promoter from *Salvia* to drive the sgRNA cassette, rather than the AtU6 promoter.

## Materials and Methods

### Plant materials, growth, and culture conditions

This study used an inbred fifth generation *S. miltiorrhiza* line originally collected in Laiwu, Shandong Province, China. The seeds of bh the 2–7 lines were surface-sterilized with 5% sodium hypochlorite, then placed on solid hormone-free MS basal medium containing 8 g/L agar. Culture conditions were maintained at 25 °C under a photoperiod of 16 h light/8 h dark. Seedlings were transferred to pots filled with 3:1 mixed soil and vermiculite at the same temperature and light conditions in a culture room.

### SgRNA design and vector construction

For the *SmCPS1* gene, three sgRNAs were designed as targets. Basic vectors contained both the Cas9 and the sgRNA expression cassettes driven by, respectively, the CaMV 35S promoter and AtU6 promoter, both of which were cloned from *Arabidopsis thaliana* and were kindly supplied by Jian-Kang Zhu from the Shanghai Center for Plant Stress Biology, Chinese Academy of Sciences. The AtU6-26SK vector was digested by *Bbs*I. Then, the designed 20 bp targeting sequence was cloned using the *Bbs*I site. After that, the AtU6-26SK vector (containing the targeting sequence) was double digested with *Kpn*I and *Sal*I (Figure S1A). The 35S-Cas9-SK vector was double digested with *Sal*I and *EcoR*I, while the pCambia1300 vector (Cambia, Canberra, Australia) was double digested with *Kpn*I and *EcoR*I. Finally, the sgRNA (small guide RNA) expression cassette between *Kpn*I and *Sal*I, together with the *Sal*I and *EcoR*I fragment of the Cas9 expression cassette were cloned into the pCambia1300 vector for stable transformation of *S. miltiorrhiza* (Figure S1B). *E. coli* competent cells and the Accc10060 *A. rhizogenes* strain were produced in our laboratory.

### *Agrobacterium*-mediated transformation and growth medium

We used a previously-reported method (Yang and Wang, 2007) with slight modifications. Briefly, for *A. rhizogenes*-mediated transformation, single colonies of *A. rhizogenes* Accc10060 harboring different CRISPR/Cas9 vectors were inoculated into 10 mL of liquid YEB medium with 50 mg/L kanamycin and 100 mg/L rifampicin, and then cultured in a shaker at 28 °C at 180 rpm for 16–18 h. Cells were collected by centrifugation when the OD600 reached 0.6, and were re-suspended in liquid MS medium until the OD600 reached 0.3. Leaves were cut into 0.5 × 0.5 cm^2^ discs and pre-cultured for 2 days on MS basal medium. The discs were submerged by shaking in a bacterial suspension for 15 min and were then co-cultured on the same medium for 2 days. The leaf discs were then transferred to selection medium (MS basal medium containing 5 mg/L hygromycin and 225 mg/L timentin). When the hairy roots were 2~3 cm in length, they were excised and cultured on solid, hormone-free MS basal medium (with 5 mg/L hygromycin and 225 mg/L timentin but no ammonium nitrate) and were subsequently maintained at 25 °C in the dark and were routinely subcultured every 25–30 days.

### Silver-stained denaturing PAGE separation and Genotype analysis

DNA extraction from 1.5-month old hairy roots was carried out following a previously-described method using CTAB^29^,^30^. Primers were designed to amplify a region of about 150 bp of *SmCPS1* containing target sites (Table S3), followed by silver-stained denaturing PAGE separation^26^. PCR products were denatured in a thermocycler at 94 °C for 5 min with loading buffer and were then cooled immediately on ice. PAGE gels consisted of 6% polyacrylamide, 0.420 g/ml carbamide, 5X TBE buffer, 0.6 μl/ml TEMED, and 3 μl/ml ammonium persulfate, and were 48 × 35 cm^2^ in size. The gel was run at a constant power of 85 W until the bands were well separated, and were then placed into 1.2 L of 10% acetic acid on a shaker for 20 min. Afterwards, the gel was dyed with silver buffer on a shaker for 30 min and developed in developing liquid (36 g sodium carbonate, 1.2 L water, 1.8 ml formaldehyde, and 200 μl sodium thiosulfate). The PCR products that only had one band on the gel were sequenced directly; others products were cloned into the pGEM-T easy vector (Promega); plasmids from individual colonies were sequenced by the Meiji company in Beijing, China.

### Metabolite analysis

100 mg of fresh hairy roots that had been cultured for 2 months were vacuum freeze dried and then frozen in liquid nitrogen and ground to a fine powder under continuous cooling. The powder was extracted in 1.5 mL of methanol that contained an internal standard (umbelliferone 20 mg/L). The extracts were sonicated twice for 15 min, centrifuged (3,000 rpm) for 10 min, and then filtered through a 0.2 μm PTFE syringe filter (Agilent).

Samples were analyzed by LC-qTOF-MS^17^,^31^. Briefly, analyses were conducted using an Agilent 1290 Infinity UHPLC system coupled to an Agilent 6540 Q-TOF LC-MS system equipped with a dual electrospray ion source operated in positive mode. 5 μl of the extracts was separated on an Agilent ZORBAX RRHD SB-C18 column (2.1 × 100 mm, 1.8 μm). Chromatographic separation was performed over a 15 min analysis time using an organic mobile phase (0.1% (v/v) formic acid in acetonitrile) as solvent A and an aqueous mobile phase (0.1% (v/v) formic acid in deionized water) as solvent B following a linear gradient. The gradient was: linear gradient from 10% to 20% A (0–5 min), linear gradient from 20% to 40% A (5–7 min), linear gradient from 40% to 100% A (7–10 min), isocratic at 100% A (10–14 min), and linear gradient from 100% to 10% A (14–15 min). The flow rate of the gradient mobile phase was 0.25 ml/min, and the column temperature was 30 °C. The mass spectrometry conditions were as follows: scan range from 100 to 1000 m/z, drying gas temperature of 350 °C, drying gas flow of 10 L/min, nebulizer pressure of 40 psi, capillary voltage of 4000 V, the sheath gas of 11 L/min at temperature of 350 °C. The Q-TOF acquisition rate was set to 0.5 s.

To measure the levels of cryptotanshinone, tanshinone IIA, and tanshinone I, extracts were analyzed with a UPLC-MS/MS systems consisting of an Agilent 1290 Infinity LC pump and a 6460 triple quadrupole mass spectrometer. The positive mode analysis was performed in multiple reaction monitoring mode (MRM). The mass spectrometry parameters were optimized with standards for each of the analyte metabolites. The precursor/product ion of cryptotanshinone is 297 > 254.1; tanshinone IIA is 295 > 266.1; tanshinone I is 277 > 262.1. An Agilent ZORBAX RX-SIL column (10032.1 mm, 1.8 μm) was used, and the column temperature was maintained at 40 °C. Solvent A was acetonitrile, and solvent B was 0.1% formic acid (v/v) in water. The separation method had a flow of 0.40 mL/min: 0 to 1.5 min, 60% solvent A; 1.5 to 3 min, a linear gradient to 80% of A; 3 to 4 min, a linear gradient to 90% of A; 4 to 5 min, 100% of A; 5 to 6 min, a linear gradient to 60 of A and keep for 1 min. The ion source parameters were set as follows: drying gas temperature of 325 °C (nitrogen), drying gas flow of 7 liters/min, nebulizer of 40 psi, sheath gas heater of 350 °C, sheath gas flow of 12 L/min, capillary voltage of 3 kV. The concentrations were quantified based on standard curves prepared with authentic reference standards.

## Additional Information

**How to cite this article:** Li, B. *et al*. Targeted mutagenesis in the medicinal plant *Salvia miltiorrhiza. Sci. Rep.* **7**, 43320; doi: 10.1038/srep43320 (2017).

**Publisher's note:** Springer Nature remains neutral with regard to jurisdictional claims in published maps and institutional affiliations.

## Supplementary Material

Supplementary Information

## Figures and Tables

**Figure 1:**
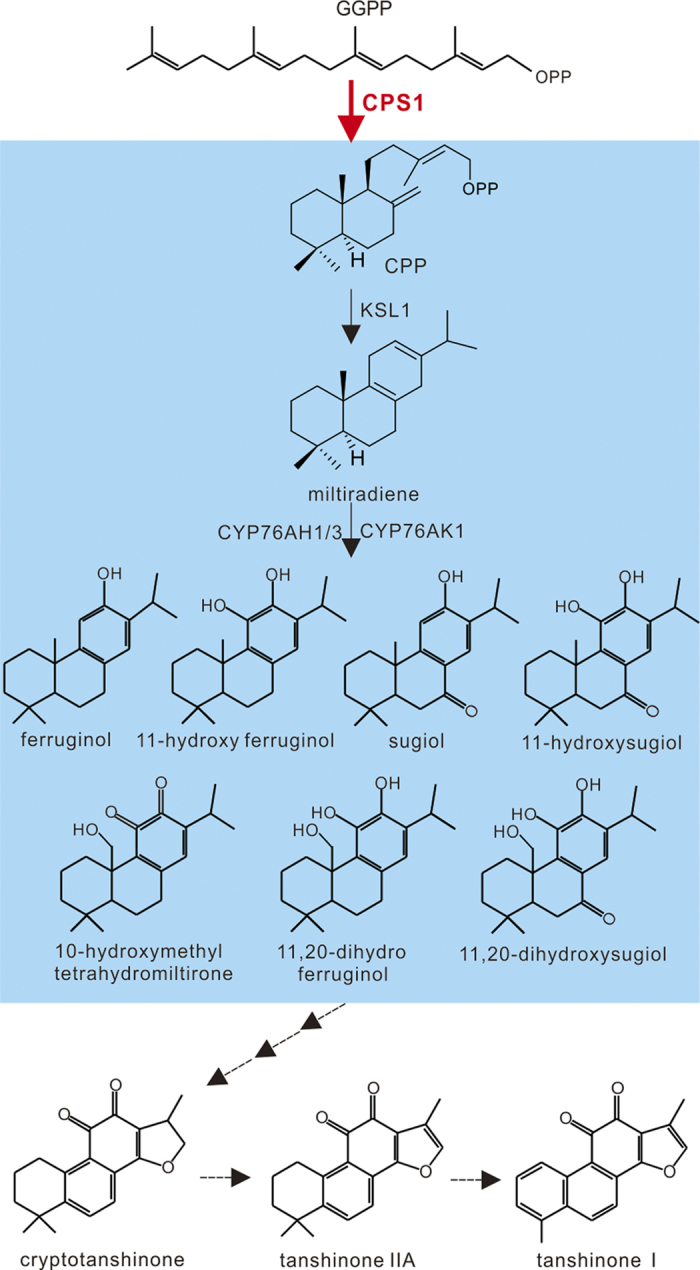
The tanshinone biosynthetic pathway in *Salvia miltiorrhiza*. The predicted biosynthetic pathway of tanshinones. SmCPS1 is the entry enzyme, and uses the precursor of diterpenes, GGPP, as its substrate for generating tanshinones. Solid arrows indicate established relationships; dashed arrows indicate hypothetical relationships.
