# Supplementary material for: Targeted mutagenesis in the medicinal plant Salvia miltiorrhiza
Source: Sci Rep. 2017 Mar 3;7:43320. doi: 10.1038/srep43320 (PMC5335714; doi:10.1038/srep43320)
Supplement: Supplementary Information [file srep43320-s1.pdf]

Targeted mutagenesis in the medicinal plant *Salvia miltiorrhiza*

Bin Li[1,2], Guanghong Cui[3], Guoan Shen[2], Zhilai Zhan[3], Luqi Huang[3], Jiachun Chen[1,*] and Xiaoquan Qi[2,*]

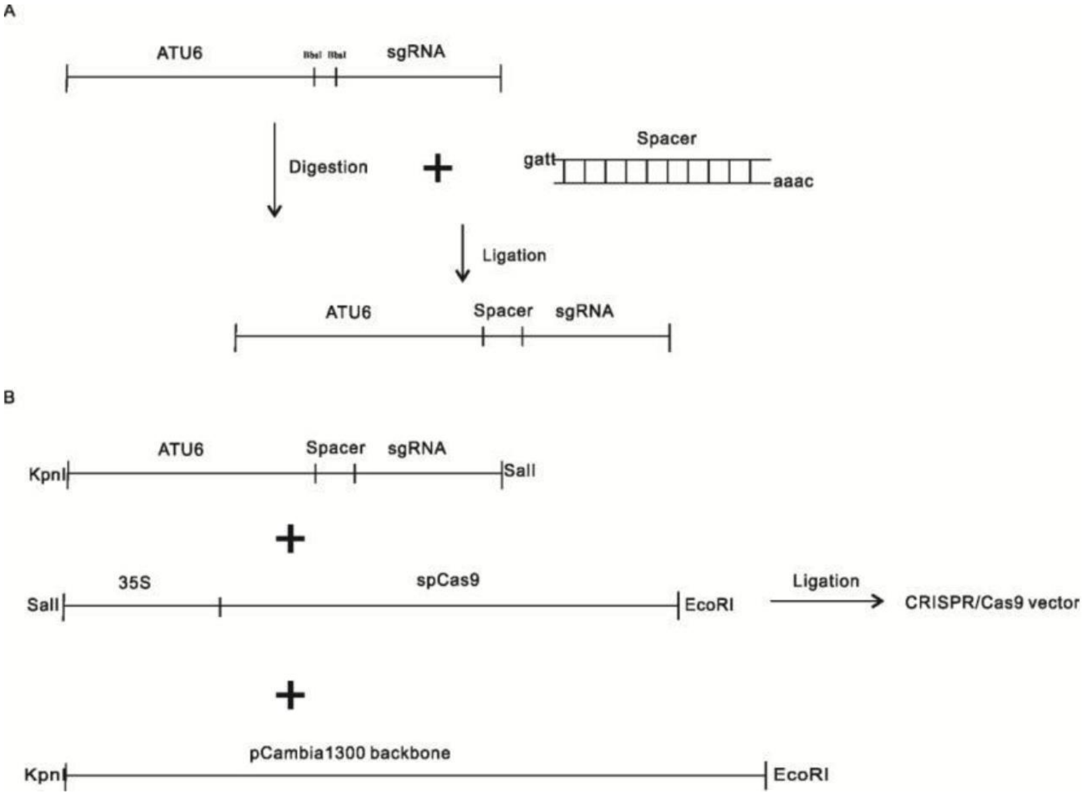

Figure S1. Vector construction process of CRISPR/Cas9. (A) The spacer is cloned into ATU6-26SK to form ATU6-sgRNA by digestion and ligation. (B) The sgRNA expression cassette between KpnI and SalI together with the SalI and EcoRI fragment of the Cas9 expression cassette are cloned into pCambia1300 vector for stable transformation of *S. miltiorrhiza*.

Table S1 Statistics of hairy root mutants.

	Number of root lines	Number of transgenic lines	Number of mutants	Number of Hm	Number of Cm
sgRNA1	36	20	0*	0*	0*
sgRNA2	40	24	0*	0*	0*
sgRNA3	48	26	11	3	8

*No genome editing process happened in sgRNA1 and sgRNA2. Hm, homozygous mutant, Cm, chimeric mutant.

Table S2 Statistic of different indel sizes in each chimeric mutant by single colony sequencing.

Chimeric mtant	Indel	Frequency of E.coli clones
No.2	TGGTACGAGAGCTGCGGCATCGAGGAA------------------GGA	10
	TGGTACGAGAGCTGCGGCATCGAGGAA-T-----------------GA	4
No.4	TGGTACGAGAGCTGCGGCATCGAGGAAATTCGGGATAAGCAGAAAGGA	2
	TGGTACGAGAGCTGCGGCATCGAG----TTCGGGATAAGCAGAAAGGA	5
	---------------GG-------------------------------	3
	TGGTACGAGAGCTGCGGCATCGA-----TTCGGGATAAGCAGAAAGGA	4
No.10	TG--------GGAGCATCTTCGA-------------------------	8
	TGGTACGAGAGCTGCGGCATCGAGGAA-T-CGGGATAAGCAGAAAGGA	16
No.35	TGGTACGAGAGCTGCGGCATCGA-----TTCGGGATAAGCAGAAAGGA	11
	TGGTACGAGAGCTGCGGCATCGAG----TTCGGGATAAGCAGAAAGGA	6
	TGGTACGAGAGCTGCGGCATCGA---------GG---------AAGGA	1
No.37	TGGTACGAGAGCTGCGGCATCGAGG--------GATAAGCAGAAAGGA	8
	TGGTACGAGAGCTGCGGCATCGAGGAATTTCGGGATAAGCAGAAAGGA	5
	TGGTACGAGAGCTGCGGCATCGAGGAA-T-------AAGCAGAAAGGA	6
No.38	TGGTACGAGAG------------------TCGGGATAAGCAGAAAGGA	5
	TGGTACGAGAGCTGCGGCATC-------TTCGGGATAAGCAGAAAGGA	7
No.40	TGGTACGAGAGCTGCGGCATCGAGGA--TTCGGGATAAGCAGAAAGGA	15
No.48	TGGTACGAGAGCTGCGGCATCGAGG---TTCGGGATAAGCAGAAAGGA	10

Table S3 List of primers used in this study.

Usage	Primer name	Sequence（5‘-->3’）
Target oligo	CPS1-SPACER1-F	GATTCGGCGTCGGCTAAGCTTCAC
	CPS1-SPACER1-R	AAACGTGAAGCTTAGCCGACGCCG
	CPS1-SPACER2-F	GATTGTGCAGAATCAACTCGAGGA
	CPS1-SPACER2-R	AAACTCCTCGAGTTGATTCTGCAC
	CPS1-SPACER3-F	GATTGCTGCGGCATCGAGGAATTC
	CPS1-SPACER3-R	AAACGAATTCCTCGATGCCGCAGC
PCR	M13-F	CGCCAGGGTTTTCCCAGTCACGAC
	M13-R	AACAGCTATGACCATG
	35S-CAS9-F	GACAAGAAGTACAGCATCGGCC
	35S-CAS9-R	ATGTCGCTCAGCAGGATGGC
	CPS1-CRISPR3-T-F	GCGATGCATGGGGAGCTCGTTA
	CPS1-CRISPR3-T-R	AGCTCGAAGATGCTCGCGGT
	CPS1-CRISPR1-F	CCCGTTCCGCCACTGGTCAT
	CPS1-CRISPR1-R	CCGCATCATGGCCTTGATGA
	CPS1-CRISPR2-F	CGGGGGACGGGAGAATAAGC
	CPS1-CRISPR2-R	GCGCCGGAAACACCACTTCG
	CPS1-CRISPR3-F	CCGCGTCGAGGCGAAGTACTACAT
	CPS1-CRISPR3-R	GCGCCGTTTGTGTCGTTGAG

Data S1 Program for potential off-target sites analysis.

```
#/usr/bin/perl

#A example for usage:perl find_match4b.pl scaffold_v3.final.scaffolds.fasta

CGGCGTCGGCTAAGCTTCACCGG

die "Usage: perl $0 GenomeSequenceFile sgRNAsequence\n" unless (@ARGV == 2);

use Cwd;

$file="scaffold_v3.final.scaffolds.fasta";#genome example

$file=$ARGV[0];

$path="C:/Users/shenga/Desktop/shenguoan20150926/libin";#path example

$path = getcwd;

if ($path=~/\\/){$path=~s/\\/\//g;};

$Seqstring="CGGCGTCGGCTAAGCTTCACCGG";#sgRNA example

$Seqstring=$ARGV[1];

@Seqstring=split(//,$Seqstring,);

$SeqstringNum=@Seqstring;

open (SeqFile, "$path/$file") or die "Can not open file $ARGV[0]\n";

open (SeqFileOUT1, ">$path/$file.txt") or die "Can not open file $ARGV[0].txt\n";

print SeqFileOUT1  "sgRNA sequence\tChrname\tOfftarget sequence\tMismatched Num\tMismatch
type\n";

local $/="\n>";

while(<SeqFile>){

        chomp;

        s/>//;
```

```
		($chrname, $sequence) = split /\n/,$_,2;
		$sequence=~s/\s+//g;
		$expectedNum=1;
		while ($expectedNum<=4) {
			$k1=0;
			while ($k1<$SeqstringNum) {
				@Seqstring2=@Seqstring;
				$Seqstring2[$k1]="[ATCG]";
				if ($expectedNum==1){
					$query=join("",@Seqstring2);
					if (@matched_result=$sequence=~/($query)/g)
{$resultNum=@matched_result;print SeqFileOUT1
"$query\t$chrname\t@matched_result\t$resultNum\tone mismatch\n";};
					};
				if ($expectedNum>1){$k2=$k1+1;}else {$k2=$SeqstringNum};
				while ($k2<$SeqstringNum) {
					@Seqstring3=@Seqstring2;
					$Seqstring3[$k2]="[ATCG]";
					if ($expectedNum==2){
						$query=join("",@Seqstring3);
						if (@matched_result=$sequence=~/($query)/g)
{$resultNum=@matched_result;print SeqFileOUT1
"$query\t$chrname\t@matched_result\t$resultNum\ttwo mismatches\n";};
```

```
};

if ($expectedNum>2){$k3=$k2+1;}else
{$k3=$SeqstringNum};

while ($k3<$SeqstringNum) {

@Seqstring4=@Seqstring3;

$Seqstring4[$k3]="[ATCG]";

if ($expectedNum==3){

$query=join("",@Seqstring4);

if
(@matched_result=$sequence=~/($query)/g) {$resultNum=@matched_result;print SeqFileOUT1
"$query\t$chrname\t@matched_result\t$resultNum\tthree mismatches\n";};

};

if ($expectedNum>3){$k4=$k3+1;}else
{$k4=$SeqstringNum};

while ($k4<$SeqstringNum) {

@Seqstring5=@Seqstring4;

$Seqstring5[$k4]="[ATCG]";

$query=join("",@Seqstring5);

if
(@matched_result=$sequence=~/($query)/g) {$resultNum=@matched_result;print SeqFileOUT1
"$query\t$chrname\t@matched_result\t$resultNum\tfour mismatches\n";};

$k4++;

};
```

```
                        $k3++;
                        };
                    $k2++;
                    };
                $k1++;
                };
        $expectedNum++;};
    };
local $/="\n";
close (SeqFile);
close (SeqFileOUT1);
```
